# Physics-Informed Genetic Optimization for Near-Field Beam Shaping in Phased Array Radar Sensing

**DOI:** 10.3390/s26144573

**Published:** 2026-07-19

**Authors:** Benzion Levy, Lior Maman, Amir Boag, Ely Levine, Yosef Pinhasi

**Affiliations:** 1 Faculty of Engineering, Ariel University, Ariel 40700, Israel; yosip@ariel.ac.il; 2 Faculty of Engineering, Tel Aviv University, Tel Aviv 69978, Israel; 3 Faculty of Engineering, Afeka College, Tel Aviv 6910717, Israel

**Keywords:** phased array, genetic algorithm, near-field, far-field, Fresnel, array factor, array pattern, phase shifter

## Abstract

Near-field beam shaping for phased-array antennas operating in the Fresnel region is a challenging non-convex electromagnetic synthesis problem, requiring coherent control of the radiated fields while accounting for the distinct positions, radiation patterns, and polarization states of individual array elements. This paper presents a physics-informed optimization framework for near-field beam shaping based on a unified vector formulation that enables the direct coherent summation of the electromagnetic fields radiated by array elements despite their distinct local spherical coordinate systems. Unlike conventional formulations that rely on repeated transformations between local spherical and global Cartesian coordinate systems, the proposed representation preserves the physical polarization properties of the electromagnetic field while providing a rigorous framework for near-field beam synthesis. To optimize the electromagnetic energy distribution over finite target surfaces rather than a single focal point, an analytical near-field point-focusing solution is integrated into the optimization process through a physically informed initialization strategy. The resulting non-convex optimization problem is solved using a genetic algorithm (GA) to determine the element phase distribution that maximizes electromagnetic energy within the prescribed target region while minimizing undesired field leakage. The proposed methodology is validated through full-wave electromagnetic simulations and extensive experimental measurements using a dedicated phased-array platform, including the design, fabrication, characterization, and calibration of the antenna array and phase-control network. The results demonstrate flexible near-field beam shaping and controlled energy focusing over finite target regions. The proposed framework is applicable to biomedical radar sensing, near-field synthetic aperture radar (SAR) illumination, wireless power transfer (WPT), high-power microwave (HPM) systems, and near-field millimeter-wave communications.

## 1. Introduction

Phased-array antennas provide flexible electromagnetic field control through the independent excitation of multiple radiating elements and are widely employed in radar, wireless communications, sensing, and imaging systems [1,2,3,4]. While conventional phased-array synthesis has traditionally focused on far-field beam steering, many emerging applications—including biomedical radar sensing, near-field synthetic aperture radar (SAR), wireless power transfer (WPT), and high-power microwave (HPM) systems—require precise control of the electromagnetic energy distribution within the near-field (Fresnel) region [5,6,7,8].

Unlike far-field beam steering, where the radiated fields are approximated as plane waves and linear phase gradients determine the beam direction, near-field beam synthesis requires coherent superposition of spherical wavefronts to shape the electromagnetic field within a finite spatial region [5]. Classical near-field focusing is typically achieved by compensating the propagation delay from each array element to a prescribed focal point, resulting in a spherical phase distribution across the aperture. Conformal array geometries provide one possible implementation by equalizing the propagation distances through the array geometry itself [9]. However, such configurations generally suffer from increased mechanical complexity, limited electronic reconfigurability, and fixed focusing distances. Furthermore, many practical applications require the illumination of extended target surfaces rather than a single focal point, making simple geometric focusing insufficient.

Optimization-based electromagnetic field synthesis has recently attracted considerable attention in applications such as programmable metasurfaces, range–Doppler signature manipulation, SAR image deception, and controllable scattering through phase modulation [10,11,12,13]. Related optimization techniques have also been investigated for phased-array beam synthesis, including beam broadening, transmit null steering, and near-field focusing [14,15,16,17,18,19,20]. While these studies demonstrate the effectiveness of optimization-based electromagnetic synthesis, most existing approaches either address far-field beam synthesis, focus on scattering manipulation rather than energy focusing, or neglect the rigorous vector treatment of polarization required for accurate near-field beam synthesis.

The principal contribution of this work is the development of a physics-informed mathematical framework for near-field beam synthesis based on the direct coherent summation of the electromagnetic fields radiated by array elements while explicitly accounting for their positions, radiation patterns, and polarization states. A unified vector formulation is derived that enables coherent field superposition despite the distinct local spherical coordinate systems associated with individual radiating elements. In contrast to conventional formulations, which repeatedly transform field components between local spherical and global Cartesian coordinate systems, the proposed representation preserves the physical polarization characteristics while avoiding repeated coordinate transformations, thereby providing a rigorous framework for near-field beam synthesis.

To solve the resulting non-convex beam-shaping problem, the proposed framework integrates the analytical solution of the classical near-field point-focusing problem into the optimization process through a physics-informed initialization strategy. The resulting phase-only optimization problem is solved using a genetic algorithm (GA), which determines the phase excitation of each transmitting element to maximize the electromagnetic energy over a prescribed target surface while minimizing undesired field leakage outside the region of interest. Because the optimization is initialized using an analytical focusing solution, the search is naturally guided toward physically meaningful beam configurations while retaining the flexibility required for arbitrary beam-shaping objectives.

The proposed methodology is validated through full-wave electromagnetic simulations and an extensive experimental campaign involving the complete design, fabrication, calibration, and characterization of a dedicated one-dimensional phased-array antenna and its associated phase-control network. The agreement between simulations and measurements demonstrates the practical applicability of the proposed framework for near-field beam shaping over finite target regions.

The remainder of this paper is organized as follows. Section 2 presents the theoretical formulation of the proposed vector beam-synthesis framework and the optimization methodology. Section 3 describes the numerical simulations and optimization results. Section 4 presents the experimental implementation and measurement validation using the fabricated phased-array prototype. Finally, Section 5 summarizes the main conclusions and outlines directions for future research.

## 2. Analysis of the Near-Field Dipole Array Pattern

This section develops the physics-based vector formulation that forms the theoretical foundation of the proposed near-field beam-synthesis methodology. Unlike conventional array formulations, which require transforming the radiated field of each element from its local spherical coordinate system into a common Cartesian reference frame before coherent superposition, the proposed formulation derives a unified vector representation that enables direct coherent summation of the electromagnetic fields while naturally preserving the polarization characteristics of the radiated fields.

The derivation is developed step by step, beginning with the well-established radiation characteristics of a single half-wave dipole and progressing toward a general formulation for an array of arbitrarily positioned and oriented elements. Throughout the analysis, it is assumed that the observation point, denoted by the position vector **r**, lies in the Fraunhofer region of each individual radiating element while simultaneously residing within the Fresnel region of the overall array. This assumption permits the use of the far-field expression for each element while accurately describing the near-field behavior of the complete array. The resulting formulation forms the analytical foundation for the optimization methodology presented in the following section.

### 2.1. Vector Formulation of Radiation by a Single Half-Wave Dipole

**Figure 1:**
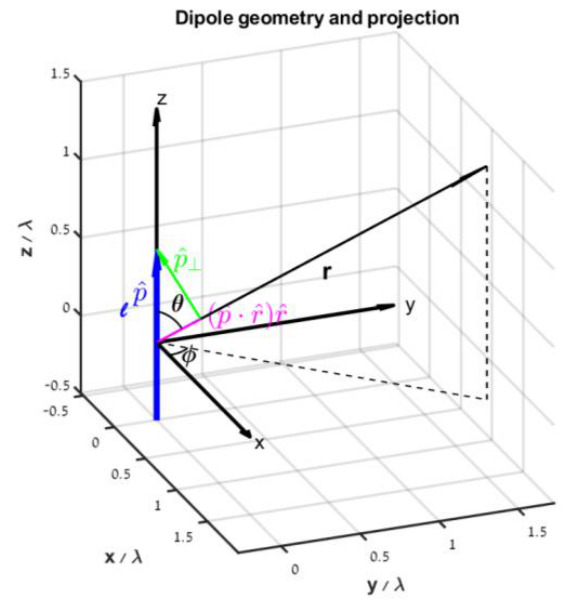
Dipole element at the origin of a coordinate system and polarization projection.

Consider a single halfwave dipole element centered at the origin, directed along the z-axis (see Figure 1), and excited by a time-harmonic current of complex amplitude $\tilde{I}$. The z-directed current is approximated by a cosine distribution within the dipole length l. The resulting electric field in the Fraunhofer (far-field, l≪r) region for a half-wave dipole (l=λ/2), is given by [21]:

(1) $$\tilde{E}\left(r,\theta \right)=-j\eta \tilde{I}\frac{e^{-jkr}}{2\pi r}\frac{\cos\left(\frac{\pi }{2}cos\theta \right)}{sin\theta }\hat{\theta }=j\eta \tilde{I}\frac{e^{-jkr}}{2\pi r}\frac{\cos\left(\frac{\pi }{2}cos\theta \right)}{{sin}^{2}\theta }e$$

where *r* and θ are the conventional spherical coordinates and k is the wavenumber, while the free-space impedance is denoted $\eta =\sqrt{\mu /\epsilon }$. To obtain a representation that can be readily generalized to arbitrarily positioned and oriented radiating elements, we introduce the vector

(2) $$e=\hat{z}-\left(\hat{z}\cdot \hat{r}\right)\hat{r}=\hat{z}-cos\theta \hat{r}=-sin\theta \hat{\theta }$$

which represents the projection of the dipole polarization onto the plane perpendicular to the propagation direction and therefore explicitly satisfies the transverse-field condition.

The vector representation introduced above can now be generalized to an array of arbitrarily positioned and oriented dipoles. Consider an array composed of radiating elements located at positions $r_{i},i=1,\ldots ,N$ and oriented along arbitrary unit vectors ${\hat{p}}_{i}$. The polarization vector associated with the field radiated by the ith element toward the observation point r is then:

(3) $$e_{i}={\hat{p}}_{i}-\left({\hat{p}}_{i}\cdot {\hat{R}}_{i}\right){\hat{R}}_{i}$$

where we define a unit vector ${\hat{R}}_{i}=R_{i}/R_{i}$ with $R_{i}=r-r_{i}$ and $R_{i}=|R_{i}|$

(4) $${\tilde{E}}_{i}(r)=j\eta {\tilde{I}}_{i}\frac{e^{-jkR_{i}}}{2\pi R_{i}}\frac{\cos\left(\frac{\pi }{2}{\hat{p}}_{i}\cdot {\hat{R}}_{i}\right)}{1-{\left({\hat{p}}_{i}\cdot {\hat{R}}_{i}\right)}^{2}}e_{i}$$

Here, we defined ${\hat{p}}_{i}\cdot {\hat{R}}_{i}=cos\theta_{i}$, where $\theta_{i}\;$ is the local angle between the dipole axis and the vector towards the observation point. Expression (4) obviously reduces to (1) for the above considered case (as in Figure 1) of a half-wave dipole located at the origin ($r_{i}=0\;and\;thus\;R_{i}\;replaced\;by\;r$) and oriented along the z-axis (${\hat{p}}_{i}=\hat{z}$ and thus $\theta_{i}=\theta$ and ${\hat{p}}_{i}\cdot {\hat{R}}_{i}=cos\theta$).

Equation (4) retains the same analytical structure as (1), differing only in the element position and polarization vector. Consequently, all radiating elements are described by a single analytical expression irrespective of their position or orientation, eliminating the need for repeated coordinate transformations during array–field synthesis.

Since every element is represented by the unified expression (4), the total radiated field is obtained directly through coherent superposition of the individual contributions. We use the result to find the near–field radiation pattern of a set of dipoles comprising an array. Controlling each element’s current excitation ${\tilde{I}}_{i}$, we examine the resulting beam formation and develop an optimization technique for the beam focusing in the near-field regime.

### 2.2. Array of Halfwave Dipoles

**Figure 2:**
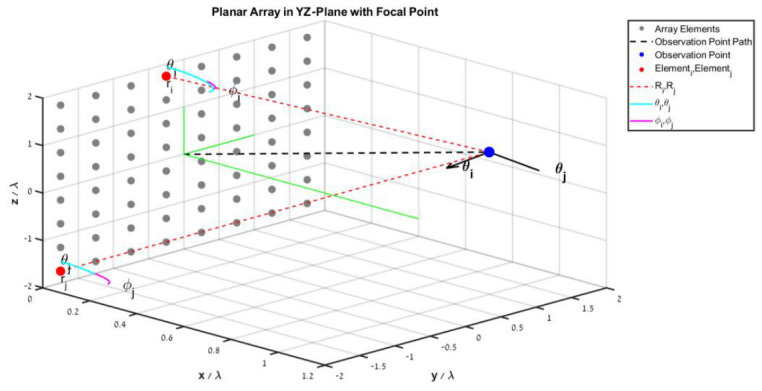
Antenna Array with 8 × 8 Elements.

Consider a two-dimensional array of dipoles, as depicted in Figure 2. The total electric field at an observation point r resulting from multiple M×N elements located at positions $r_{i}$, each with excitation ${\tilde{I}}_{i}$ and polarization $\hat{p}$, is given by a coherent summation of the individual element fields given in (5):

(5) $${\tilde{E}}_{Array}\left(r\right)=\sum_{i}{\tilde{E}}_{i}\left(r\right)=\sum_{i}j\eta {\tilde{I}}_{i}\frac{e^{-jkR_{i}}}{2\pi R_{i}}\frac{\cos\left(\frac{\pi }{2}\hat{p}\cdot {\hat{R}}_{i}\right)}{1-{\left(\hat{p}\cdot {\hat{R}}_{i}\right)}^{2}}e_{i}$$

Equation (5) constitutes the fundamental near-field beam-synthesis model used throughout the remainder of the paper.

We note that in the far–field region, where $\left|r\right|\gg \left|r_{i}\right|$, the distance $R_{i}$ can be approximated as follows:

(6) $$R_{i}=\left|r-r_{i}\right|\approx r\left[1-\frac{r_{i}\cdot \hat{r}}{r^{2}}+\frac{r_{i}^{2}}{{2r}^{2}}\right]\approx r-r_{i}\cdot \hat{r}$$

Furthermore, for an array of half-wave dipoles directed along the *z*-axis, under the far-field assumption, ${\hat{p}}_{i}\cdot {\hat{R}}_{i}=cos\theta$ and the projection vector (3) becomes identical for all the elements, i.e., $e_{i}=-sin\theta \hat{\theta }$. The total electric field for the array far-field is reduced to the well-known separable form:

(7) $${\tilde{E}}_{Array\_FF}\left(r\right)\approx \hat{\theta }j\eta \frac{e^{-jkr}}{2\pi r}\frac{\cos\left(\frac{\pi }{2}cos\theta \right)}{sin\theta }\sum_{i}{\tilde{I}}_{i}e^{-jkr_{i}\cdot \hat{r}}$$

In contrast to the far-field formulation in (7), which can be expressed as the separable product of the element radiation pattern and the array factor (AF), the near-field expression in (5) is inherently non-separable. Each array element possesses its own propagation distance, propagation direction, polarization projection, and effective radiation pattern, all of which depend on the observation point. Consequently, the individual field contributions cannot, in general, be represented by a common element pattern multiplied by a single array factor. These element-specific variations play a fundamental role in near–field beam synthesis and must be explicitly accounted for when designing the excitation phases for a desired spatial field distribution. The analytical formulation developed above therefore provides the forward electromagnetic model that forms the foundation of the optimization framework presented in the following section.

## 3. Near-Field Beam Shaping and Optimization

### 3.1. Focal Point Focusing and Coherent Integration

Before addressing beam shaping over an extended target, we first consider the canonical problem of near-field focusing at a single spatial point. This problem admits a closed-form analytical solution and provides the physical interpretation of coherent field synthesis and the initialization strategy used later in the optimization algorithm. The corresponding phase–conjugation solution maximizes the coherent superposition of the array fields at the focal point $r_{f}$ and therefore represents the theoretical upper bound on the achievable focal gain for a given aperture.

Assume that the elements lie on the planar YZ surface at x=0:

$$r_{mn}=\left(0,y_{m},z_{n}\right)\in R^{3}$$

The desired focus point is as follows:

$$r_{f}=\left(x_{f},y_{f},z_{f}\right)\forall x_{f}>0$$

The electric field at point $r_{f}$ is as follows:

(8) $${\tilde{E}}_{Array}\left(r_{f}\right)=\sum_{i}{\tilde{E}}_{i}\left(r_{f}\right)=\frac{j\eta }{2\pi }\sum_{m=0}^{M-1}\sum_{n=0}^{N-1}{\tilde{I}}_{mn}\frac{1}{\left|r_{f}-r_{mn}\right|}\frac{\cos\left(\frac{\pi }{2}\hat{p}\cdot {\hat{R}}_{mn}\right)}{1-{\left(\hat{p}\cdot {\hat{R}}_{mn}\right)}^{2}}e^{-jk\left|r_{f}-r_{mn}\right|}e_{mn}$$

where we define $R_{mn}=r_{f}-r_{mn}$ with a unit vector ${\hat{R}}_{mn}=R_{mn}/R_{mn}$ where $R_{mn}=|R_{mn}|$. The excitation coefficient ${\tilde{I}}_{mn}$ denotes the complex excitation applied to the mn-th array element. Under the phase–only constraint adopted throughout this work, its magnitude is fixed while its phase is selected to compensate for the propagation delay between the array element and the focal point. To achieve coherent integration at the focal point, the excitation phases compensate for the propagation delay from each array element to the focal location, thereby aligning the phases of all field contributions. Consequently, Equation (9) represents the analytical phase-conjugation solution for ideal point focusing and constitutes the physics-informed initialization employed in the subsequent optimization:

(9) $${\tilde{I}}_{mn}=e^{jk\left|r_{f}-r_{mn}\right|}=e^{jkR_{mn}}$$

### 3.2. Beamwidth in the Near-Field

The analytical phase-conjugation solution derived in the previous subsection produces the smallest diffraction-limited focal spot achievable for the given aperture. In many practical applications, however, the objective is not to maximize the field at a single point but rather to distribute electromagnetic energy over an extended target surface. Achieving such a prescribed field distribution requires intentionally relaxing the perfect phase coherence associated with point focusing across the aperture. The resulting beam is therefore neither a conventional far-field angular beam nor an ideal near-field point focus, but a user-defined spatial field distribution over a finite region. Unlike the point-focusing problem, this inverse near-field beam-shaping problem generally does not admit a closed-form analytical solution and consequently requires numerical optimization.

The minimal beamwidth is bounded by the diffraction limit as follows:

$$\left(∠EL,∠AZ\right){}^{\circ}=\frac{360}{2\pi }\left(\frac{\lambda }{D_{z}},\frac{\lambda }{D_{y}}\right)$$

where $D_{z}$ and $D_{y}$ represent the effective aperture sizes in the z and y directions, respectively.

### 3.3. Optimization Problem

**Figure 3:**
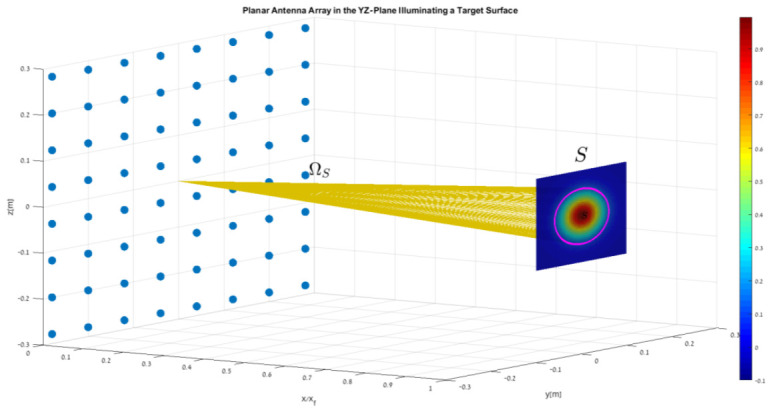
Surface S, positioned in the x-plane at a distance $x_{f}$ corresponding to the main lobe of the antenna array, illuminated by a beam with spatial beamwidth $\Omega_{S}$.

Define a two-dimensional planar surface S that is placed on the x-plane at a distance $x_{f}$ from a rectangular M×N antenna array positioned at x=0. The objective is to determine the phase-only excitation matrix $\varphi_{mn}$ that produces a prescribed electromagnetic energy distribution over the target surface. The target surface subtends the spatial angular region, see Figure 3:

$$\Omega_{S}=\left(∠EL,∠AZ\right)\in D\subset R^{2}$$

The analytical point-focusing solution of (9) serves as the physics-informed initial solution, from which the optimization relaxes perfect phase coherence to synthesize the desired spatial field distribution over the target surface. Accordingly, the beam-shaping problem is formulated as the following constrained optimization problem.

(10) $$J\left(\varphi \right)=-\frac{\iint_{{\left(\theta ,\phi \right)\in \Omega }_{S}}{\left\|{\tilde{E}}_{Array}\left(r;\varphi \right)\right\|}^{2}dS}{\iint_{{\left(\theta ,\phi \right)\notin \Omega }_{S}}{\left\|{\tilde{E}}_{Array}\left(r;\varphi \right)\right\|}^{2}dS}+\iint_{{\left(\theta ,\phi \right)\in \Omega }_{S}}{\left[\frac{\left\|{\tilde{E}}_{Array}\left(r;\varphi \right)\right\|}{\left\|{\tilde{E}}_{Array}\left(r;\varphi \right)\right\|}-1\right]}^{2}dS$$

With the following optimization problem:

(11) $$\varphi =\mathrm{argmin}J\left(\varphi \right),\;\;Subject\;to\;I_{mn}=e^{j\varphi_{mn}}\;(phase\;only\;constraints)$$

The first term maximizes the ratio between the energy delivered inside the target region and the energy radiated outside it, while the second term penalizes field non-uniformity within the illuminated surface.

The optimization is performed under phase-only excitation constraints ${\tilde{I}}_{mn}=e^{j\varphi_{mn}}$ which preserve a constant excitation magnitude while allowing independent phase control of each array element. This constraint is also consistent with practical phased-array transmitters employing saturated power amplifiers to maximize power efficiency.

### 3.4. Optimization Using a Genetic Algorithm

We have an array of M×N elements, and each element has its own phase:

$$\varphi =(\varphi_{11},\varphi_{12},\cdots ,\varphi_{MN})$$

where each phase variable $\varphi_{mn}$ is periodic and is therefore defined on the unit circle $S^{1}$, rather than on a bounded interval of the real line.

Since each excitation phase is periodic, the optimization variables collectively form the Cartesian product of MN circles, yielding the toroidal search space:

$$T^{MN}=(S^{1}{)}^{MN},$$

rather than a Euclidean vector space.

Among the various optimization techniques applicable to this non-convex problem, a genetic algorithm (GA) was selected because it naturally accommodates periodic phase variables, does not require gradient information, and is well suited for optimizing highly nonlinear objective functions over large search spaces. The resulting optimization problem is therefore defined over the toroidal manifold $T^{MN}$, making derivative-free evolutionary optimization particularly attractive.

These characteristics make conventional gradient-based optimization difficult to apply and motivate the use of derivative-free evolutionary optimization methods such as the genetic algorithm employed in this work.

### 3.5. System Requirements and Simulation Results

The proposed methodology was evaluated using an 8×8 planar dipole array with an aperture of 0.6 m × 0.6 m  operating at $f_{c}=2.35$ GHz is considered. The target consists of a parallel planar surface of dimensions 0.2 m × 0.2 m, located at $x_{f}=1$ m in the boresight direction. The objective is to determine the phase distribution that produces a prescribed electromagnetic energy distribution over the target surface.

The selected target distance lies within the Fresnel region of the array, since:

$$0.6\sqrt{\frac{D^{3}}{\lambda }}<x<\frac{2D^{2}}{\lambda }\to 0.6\sqrt{\frac{0.6^{3}}{0.125}}<x<\frac{2\cdot 0.6^{2}}{0.125}\to 0.78m<x<5.7m$$

The spatial angular extent subtended by the target surface is ∠EL,∠AZ that ideally covers the surface in the range of 1 m yields the following equation:

$$∠EL=∠AZ=2\tan^{-1}\left(\frac{\frac{\Delta y}{2}}{x_{f}}\right)=2\cdot {tan}^{-1}\left(\frac{0.2}{2\cdot 1}\right)=11.4{}^{\circ}$$

The required angular extent ($11.4^{\circ }$) is close to the diffraction-limited beamwidth achievable with the considered aperture. The minimum half-power beamwidth (HPBW) of a uniformly illuminated rectangular aperture is approximately 0.886λ/D, yielding

$$(∠EL,∠AZ{)}_{min}=\frac{360}{2\pi }\left(\frac{0.886\lambda }{D_{z}},\frac{0.886\lambda }{D_{y}}\right)\approx (10.5^{\circ },10.5^{\circ }).$$

This confirms that the selected target dimensions are close to the smallest surface that can be illuminated without violating the diffraction limit.

Unlike conventional far-field beam synthesis, where the azimuth and elevation dimensions can often be optimized independently through separable array-factor formulations, near-field beam shaping requires simultaneous optimization of the complete two-dimensional phase distribution because the electromagnetic field is inherently non-separable.

The optimization is initialized using the analytical phase-conjugation solution of (9), after which the genetic algorithm iteratively relaxes perfect coherent focusing to obtain the desired energy distribution over the finite target surface.

## 4. Experimental Validation

This section experimentally validates the proposed near-field beam-synthesis methodology using a dedicated phased-array measurement platform. The optimization algorithm itself is executed offline to determine the required excitation phases. These optimized phase distributions are then implemented using the fabricated phase-control network, and the resulting near-field radiation patterns are measured and compared with the corresponding simulations.

For experimental tractability, a one-dimensional eight-element dipole array was constructed. Although the proposed optimization framework is formulated for two-dimensional arrays, the one-dimensional implementation preserves the essential near-field focusing physics while significantly simplifying the experimental implementation, including calibration, alignment, phase control, and measurement.

The dipole element design follows the model described in Appendix A and is fully consistent with the theoretical formulation presented in Section 2.

The experimental platform consists of the dipole array, a fixed analog phase-control network based on CPW delay lines, and the associated microwave measurement hardware. The measured near-field radiation patterns are used to validate the optimized phase distributions obtained from the proposed beam-synthesis algorithm by comparing the experimental results with the corresponding numerical simulations. Because the one-dimensional array cannot independently control both spatial dimensions, the experiment validates one-dimensional near-field beam synthesis rather than full two-dimensional surface illumination.

### 4.1. Simulating a One-Dimensional Array

To illustrate the focusing behavior of the proposed near-field beam-synthesis methodology throughout the Fresnel region, simulations are first presented for focal distances of 0.5 m, 1 m, and 1.5 m. The 0.5 m case is included as an illustrative example of operation under stronger near-field conditions, where the effect of phase compensation is particularly pronounced. The subsequent experimental validation, however, is performed only for the focal distances of 1 m and 1.5 m, for which the phase-control network was designed, fabricated, and experimentally characterized.

The corresponding optimized phase distributions are then used to design and implement the experimental phase-shifter network.

Now, we simulate a one-dimensional array and use the phase shifter results to design and establish the measurement setup.

**Figure 4:**
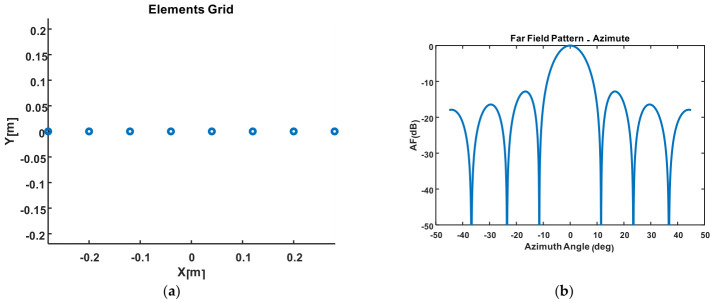

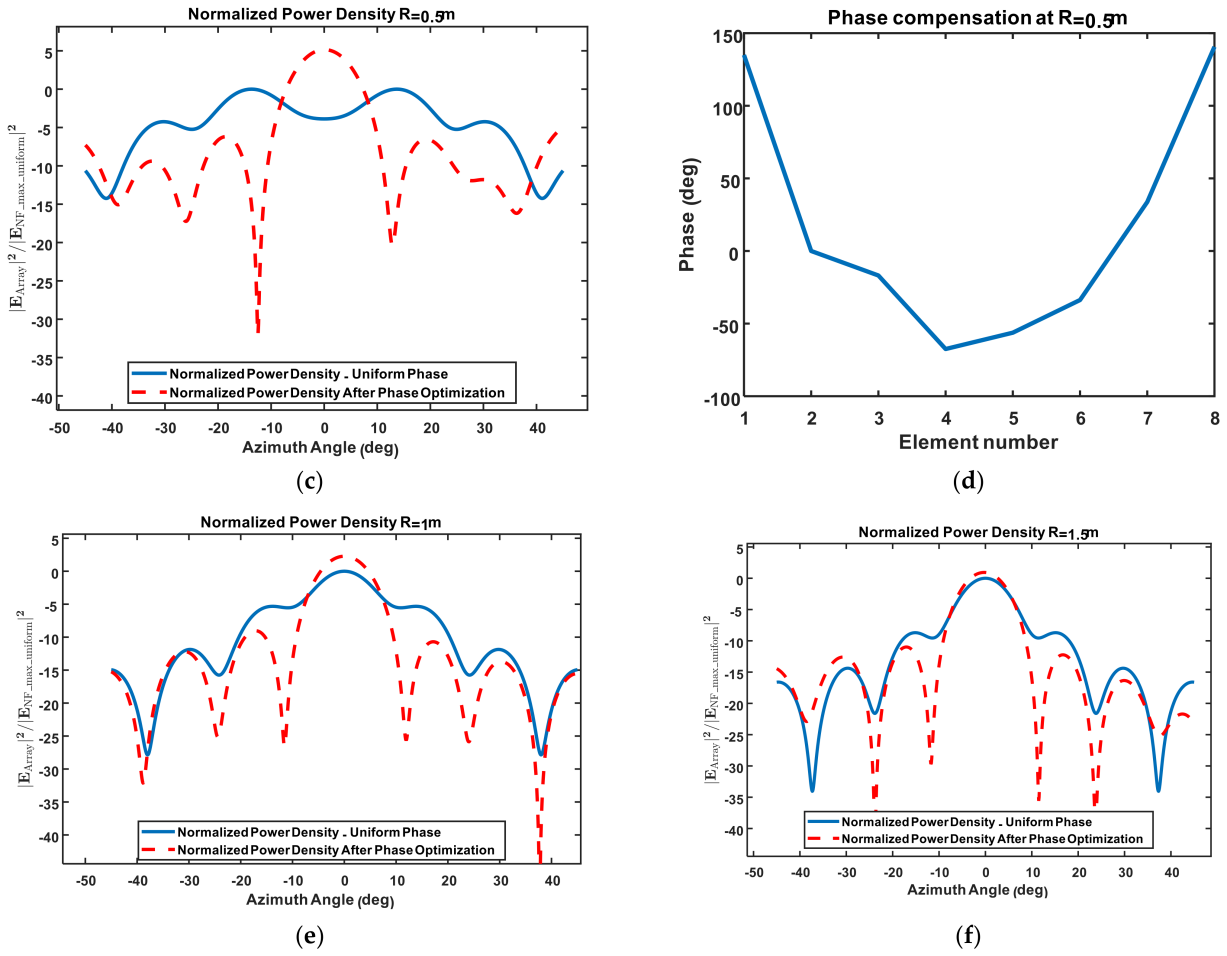
Normalized Near–Field Power Density (**a**) Element grid; (**b**) Far field pattern—Azimuth; (**c**) Normalized Near–Field Power Density with and without phase compensation at R = 0.5 m; (**d**) Phase compensation value, R = 0.5 m; (**e**) Normalized Near-Field Power Density with and without phase compensation, R = 1 m; (**f**) Normalized Near-Field Power Density with and without phase compensation at, R = 1.5 m.

Figure 4c,e,f present the normalized near-field electric-field intensity distributions, expressed as $|E{|}^{2}/|E_{max}{|}^{2}$, for focal distances of 0.5 m, 1 m, and 1.5 m, respectively. It should be emphasized that these results do not represent antenna gain or far-field radiation patterns. Instead, they depict the normalized electromagnetic field intensity, which is directly proportional to the local power density. Consequently, these plots provide a direct visualization of the energy concentration achieved by the proposed near-field beam-focusing approach.

The comparison between the uncompensated and phase–compensated cases clearly demonstrates the ability of the proposed phase compensation to concentrate the electromagnetic energy within the desired focal region. At the shortest focal distance of 0.5 m, the energy is highly localized, resulting in a narrow and well-defined focal spot with a significant increase in the peak field intensity. As the focal distance increases to 1 m and 1.5 m, the achievable energy concentration gradually decreases. This behavior is expected, since the observation point approaches the transition toward the far–field region, where the electromagnetic wavefront becomes progressively planar and the capability to manipulate the spatial field distribution through near–field phase compensation is inherently reduced.

Another notable observation is the gradual formation of pronounced nulls adjacent to the focal region. These nulls become increasingly visible as the focal distance increases and result from the coherent interference between the radiated fields of the individual array elements. Their appearance reflects the transition from a predominantly localized near-field energy distribution toward a radiation pattern that increasingly resembles the beam structure observed in the far field, characterized by a main lobe accompanied by nulls and sidelobes.

Overall, these results demonstrate that the proposed phase-compensation method effectively concentrates electromagnetic energy within the Fresnel region while illustrating the gradual reduction in spatial focusing capability as the propagation distance approaches the far-field regime.

The optimized phase distribution obtained from the algorithm was first quantized to the nearest 6-bit phase resolution in order to emulate a practical digitally controlled phased-array system. These quantized phase values were subsequently realized experimentally using fixed CPW delay lines. Since only the relative phase differences between array elements affect the focusing performance, a common phase offset was removed, yielding the phase values listed in Table 1.

### 4.2. Dipole Antenna Design

The element’s design requirements are shown in Table 2 [22]:

The dipole pre-design antenna simulation using CST 2021 software yields the results in Table A1.

### 4.3. Fixed Analog Phase-Control Network

**Figure 5:**
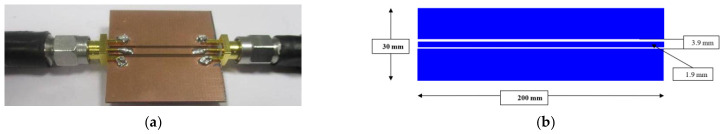
Phase shifter (**a**) Phase shifter on test (CPW) (**b**) Coplanar waveguide.

Two sets of phase shifters were built to meet the focusing goals, sharpening the beam at $x_{f}=1\;m,\;1.5\;m$ in the (∠EL,∠AZ) dimension in the desired range. This includes the fabrication of printed coplanar waveguide (CPW) transmission lines on an FR-4 substrate with SMA end-launch connectors. The printed circuit boards have a thickness of 1 mm and dimensions of 200 mm × 30 mm, with single-sided copper etching. Based on CPW transmission line simulations, when a dielectric constant of $E_{r}=4.6$ was assumed, the characteristic impedance of the line was found to be very close to 50 Ω. In addition, the phase shift was calculated to be approximately 5.7° per millimeter of line length at the operating frequency. Unlike digitally programmable phase shifters, the fabricated CPW delay lines implement only fixed phase distributions corresponding to the optimized solutions. Consequently, the experimental platform does not support dynamic beam steering or real-time beam reconfiguration. Its purpose is solely to validate the proposed near-field beam-synthesis methodology under controlled experimental conditions, see Figure 5.

### 4.4. Phase Shifter’s Set Measurements

The delay lines were designed to be $f_{c}=2.35\;GHz$, and their electric phase was computed according to the accumulated desired phase Δl=λ/2π·Δϕ. Consequently, we obtain the results in Table 3 and Table 4 and manufacture delay lines with a resolution of  0.5 mm.

**Figure A1:**
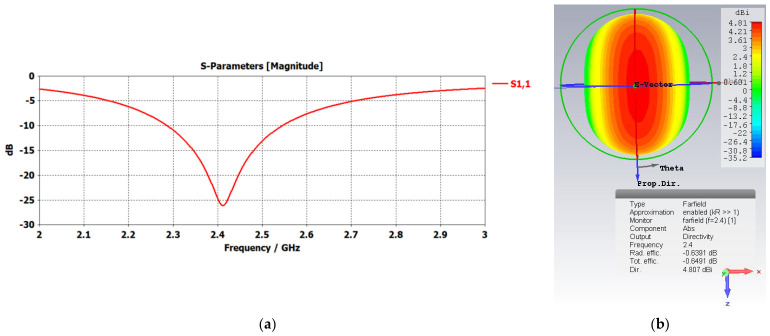

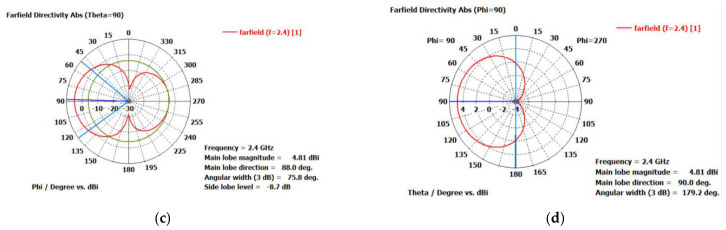
(**a**) Return loss magnitude; (**b**) far–field 3D pattern of the dipole antenna; (**c**) 2D far–field azimuth view; (**d**) 2D far–field elevation view.

As we can see from Table 3 and the measurements in Figure A1, the average phase error, including 1.3°, and the maximum phase error are 2.6°.

As shown in Table 4, the fabricated phase-shifter set exhibits an average phase error of approximately 1° and a maximum phase error of 2.6°.

**Figure 6:**
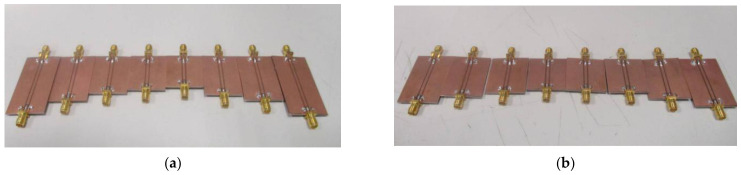
Focusing on distance (**a**) $x_{f}=1\;m$ (**b**) $x_{f}=1.5\;m$.

We can see that the delay lines have a quasi-quadradic form that is changed according to the focusing requirements. Using delay lines in the opposite order results in broadening of the beam, as explained later, see Figure 6.

### 4.5. Feeding Network

**Figure 7:**
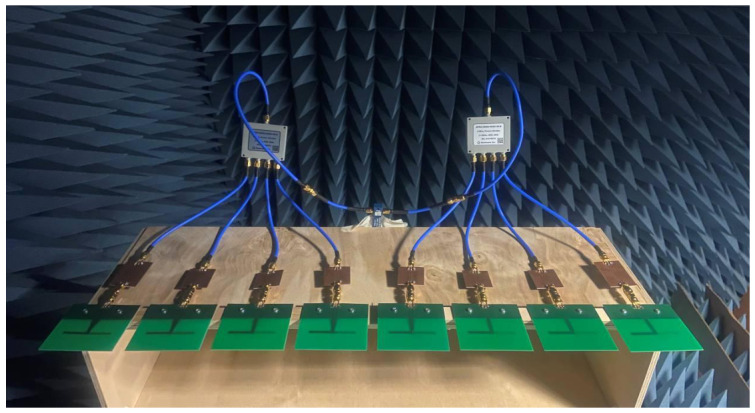
Dipole elements connected to the feeding network and phase shifters.

To feed the elements, we designed a feeding network consisting of RF cables and combiners. To preserve the relative phase between channels, identical RF cables were used. Each channel was characterized individually while all remaining channels were terminated with matched 50 Ω loads. The fabricated feeding network is shown in Figure 7.

As we can see from Table 5, the average phase error, including the feeding network, is 3.5°, and the maximum phase error is 7.7°.

To relate the experimental implementation to practical phased-array systems, we compare the achieved phase accuracy with that of a typical 6-bit digital phase shifter. A 6-bit phase resolution corresponds to a quantization step of 5.625°, yielding an estimated beam-pointing error of

$$\Delta \varphi =\frac{2\pi }{2^{n}}=\frac{2\pi }{\lambda }d_{y}sin\left(\Delta \phi \right)⟶\Delta \phi =\sin^{-1}\left(\frac{\lambda }{d_{y}}\frac{1}{2^{n}}\right)=\sin^{-1}\left(\frac{1}{2^{n-1}}\right)=1.8{}^{\circ}$$

The measured phase errors of the fabricated delay-line network are of comparable magnitude, demonstrating that the analog implementation provides phase accuracy representative of practical phased-array hardware.

Assuming that we have a digital phase shifter with a 6-bit resolution that is equivalent to a quantization error of $360/2^{6}=5.6{}^{\circ}$, the direction error of the array would be $\Delta \phi =\sin^{-1}\left(1/2^{5}\right)=1.8{}^{\circ}$. These performances are similar to the parameters in the simulation.

## 5. Measurement Results

In this section, the proposed optimization framework is experimentally validated in an anechoic chamber by implementing the optimized phase distributions and measuring the resulting antenna radiation patterns. The measured radiation patterns are then compared with the corresponding simulated optimized patterns to evaluate the accuracy of the implemented phase distribution and the resulting near-field focusing performance.

**Figure 8:**
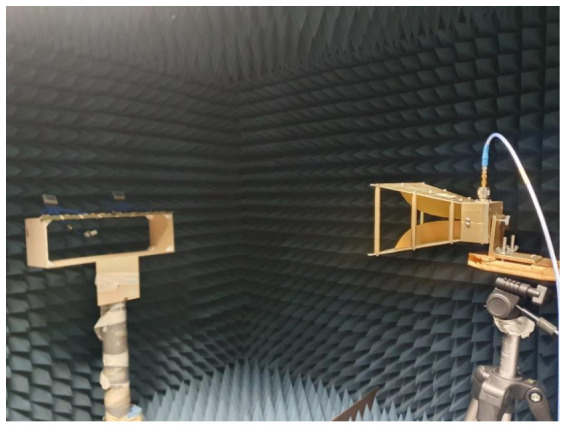
Measuring the spatial focusing beam in a chamber.

The array beam pattern was measured using the MiDAS system by mechanically rotating the transmitting antenna array while the transmitted signal was received by a separate receiving antenna, as illustrated in Figure 8. The chamber measurement configuration was limited to azimuthal rotation in the ϕ-direction only. Accordingly, both the optimization procedure and the experimental verification were conducted under this angular constraint. The MiDAS system provides real-time visualization of the received azimuth beam pattern directly from the measured signal intensities, without requiring post-processing based on Fourier-transform techniques, which are applicable primarily in the far-field regime.

**Figure 9:**
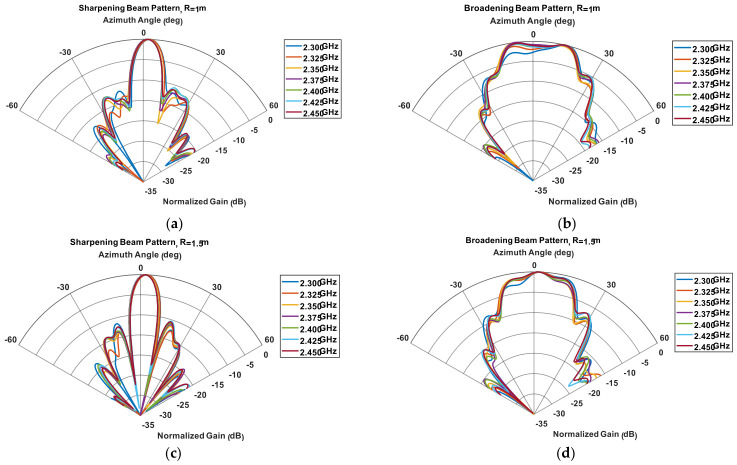
Radiation pattern measurements at frequencies of 2.3 GHz–2.4 GHz: (**a**) sharpening phase, $x_{f}=1\;m$, polar form; (**b**) broadening phase, $x_{f}=1\;m$, polar form; (**c**) sharpening phase, $x_{f}=1.5\;m$, polar form; (**d**) broadening phase, $x_{f}=1.5\;m$, polar form.

**Figure 10:**
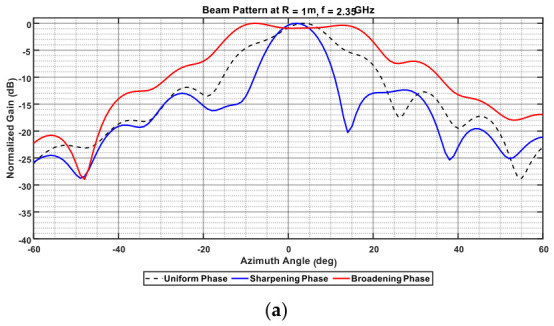

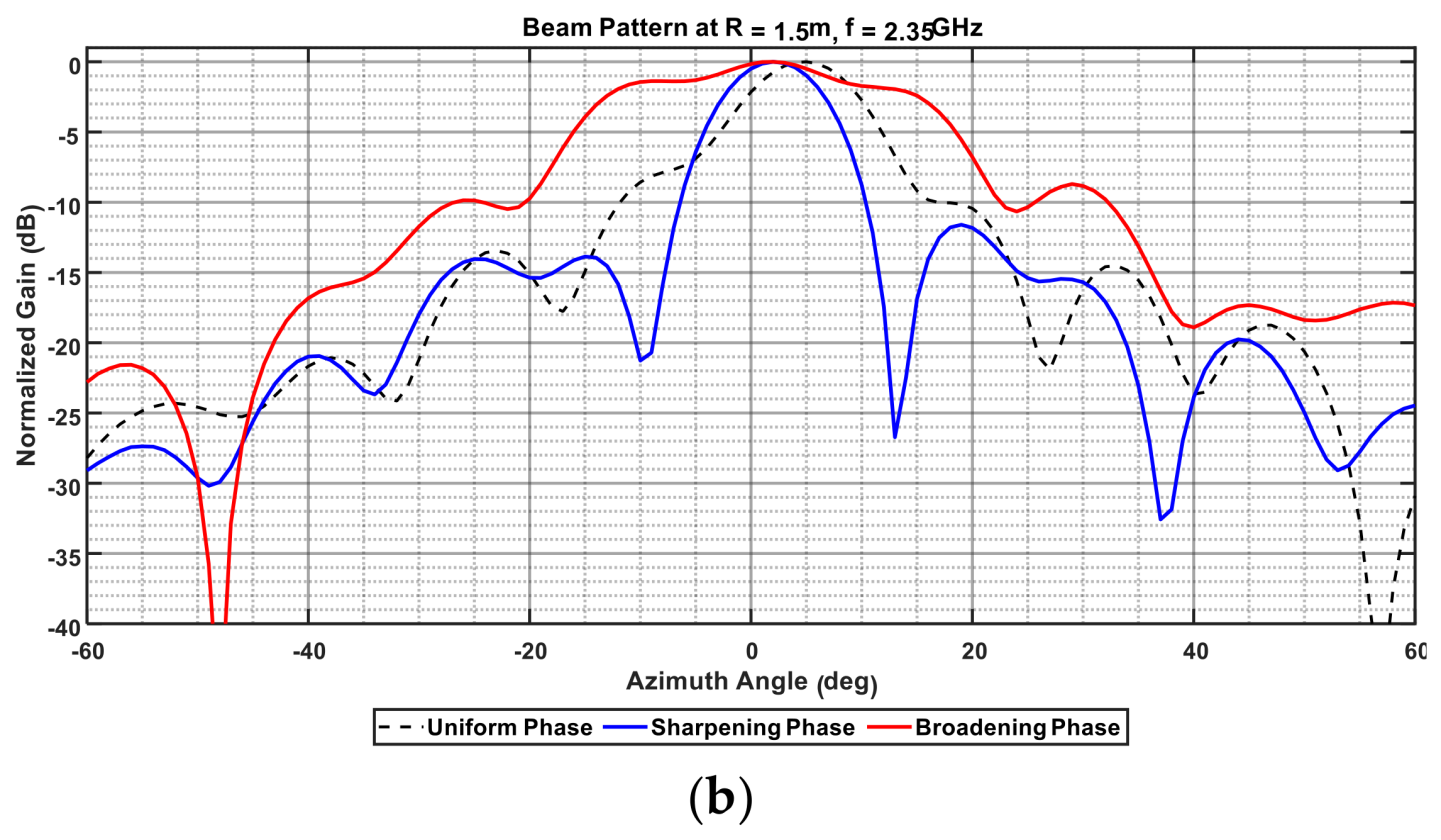
Radiation pattern measurements at frequency of 2.35 GHz (**a**) R = 1 m; (**b**) R = 1.5 m.

The measurement results presented in Figure 9 and Figure 10 demonstrate near-field beam focusing in the deep Fresnel region at focal distances of 1 m and 1.5 m, evaluated using the −3 dB beamwidth criterion. Figure 9 presents the measured azimuth radiation patterns in polar form for five uniformly spaced frequencies spanning the 2.3–2.4 GHz frequency range. Figure 9a,b correspond to the 1 m focal distance and illustrate the beam-sharpening and beam-broadening phase configurations, respectively. Similarly, Figure 9c,d present the corresponding beam-sharpening and beam-broadening radiation patterns for the 1.5 m focal distance. As expected, the beam-broadening phase distributions are approximately the inverse of the beam-sharpening phase distributions. The phase profiles producing beam focusing exhibit a concave distribution across the aperture, whereas the beam-broadening phase profiles exhibit a convex distribution.

As observed, beam sharpening was successfully achieved when the target surface was positioned at a focal distance of 1 m, yielding an illumination beamwidth of approximately $11^{\circ }$, which is close to the diffraction-limited beamwidth. In addition, the beam-broadening phase configuration produced an illumination beamwidth of approximately $30^{\circ }$, as required to fully illuminate the azimuthal dimension of the target surface.

An additional observation from Figure 9 is the high robustness of the proposed optimization algorithm with respect to frequency variations around the nominal design frequency of 2.35 GHz. The measured radiation patterns exhibit nearly identical beam-sharpening and beam-broadening characteristics over the entire 2.30–2.45 GHz frequency range, with only minor variations in beam shape and sidelobe levels. Relative to the nominal design frequency, the measurements span frequency offsets from −50 MHz to +100 MHz, introducing approximately 2–3° and 4–5° of additional phase shift, respectively, depending on the electrical length of each transmission path. Despite these phase variations, the measured radiation patterns remain highly consistent throughout the investigated frequency range. These results indicate that the proposed optimization framework is inherently robust to moderate phase perturbations of approximately 2–5° per element, confirming its suitability for practical implementations where manufacturing tolerances and operating-frequency variations inevitably introduce small phase errors.

Figure 10 presents a comparison between the uniform-phase configuration and the optimized beam-sharpening phase configuration at 2.35 GHz for two focal distances. Subplot (a) corresponds to a focal distance of $x_{f}=1\;m$, whereas subplot (b) corresponds to $x_{f}=1.5\;m$.

The measurement results reveal that the proposed optimization algorithm is most effective when operating deep within the Fresnel region. At a focal distance of $x_{f}=1\;m$, the uniform-phase excitation produces a beamwidth of approximately 15°. By applying the optimized phase distributions, the beamwidth can be intentionally narrowed to approximately 10° or broadened to approximately 30°, demonstrating the algorithm’s capability to flexibly control the spatial energy distribution over the target region.

In contrast, at a focal distance of $x_{f}=1.5\;m$, the uniform-phase beamwidth is already reduced to approximately 11°, which is close to the diffraction-limited beamwidth of the array aperture. Consequently, the optimized beam-sharpening phase distribution provides only a marginal additional reduction to approximately 10°, whereas the beam-broadening phase distribution continues to produce a beamwidth of approximately 30°.

Furthermore, the measured radiation patterns exhibit a progressively more pronounced main lobe and sidelobe structure as the focal distance increases, reflecting the gradual transition toward far-field radiation behavior. The increased visibility of the sidelobes indicates that the array response becomes increasingly dominated by the aperture diffraction characteristics.

These results demonstrate that the proposed optimization framework provides the greatest beam-shaping flexibility within the deep near-field region, where the additional spatial degrees of freedom enable significant manipulation of the electromagnetic field distribution. As the focal distance approaches the paraxial region and eventually the far-field limit, the achievable beamwidth naturally converges toward the diffraction limit imposed by the finite aperture. Consequently, the ability of the optimization algorithm to further reduce the beamwidth diminishes, not because of a limitation of the optimization itself, but because the radiation pattern is fundamentally constrained by the physical diffraction limit of the array aperture. Conversely, beam broadening remains achievable by intentionally reducing the coherent field addition across the aperture, allowing controlled redistribution of the radiated energy over a wider angular region.

## 6. Summary, Discussion and Conclusions

In this paper, we presented a physics-informed framework for near-field beam synthesis in phased-array antennas operating in the Fresnel region. The proposed formulation enables coherent vector summation of the electromagnetic fields radiated by individual array elements while explicitly accounting for their positions, orientations, radiation patterns, polarization states, and the geometry of the receiving surface. In addition, the analytical solution of the classical near-field point-focusing problem was incorporated into the optimization process through a physics-informed initialization strategy, providing a physically meaningful starting point for solving the more general beam-shaping problem over extended target surfaces.

The resulting non-convex phase-only optimization problem was solved using a genetic algorithm (GA), which served as a practical optimization tool rather than the principal scientific contribution of this work. The proposed methodology enables application-driven near-field beam synthesis by optimizing the phase excitation of each transmitting element to maximize the electromagnetic energy delivered to a prescribed target region while minimizing undesired field leakage outside the region of interest. Owing to the generality of the proposed formulation, alternative optimization techniques may also be employed without modifying the underlying electromagnetic model.

The proposed framework was validated through full-wave electromagnetic simulations together with a dedicated experimental campaign involving the design, fabrication, calibration, and characterization of a one-dimensional phased-array prototype and its associated analog phase-control network. The agreement between simulations and measurements demonstrates the practical feasibility of the proposed methodology and validates its capability to synthesize controlled near-field energy distributions.

Potential applications of the proposed framework include biomedical radar sensing of respiration and cardiac activity in the millimeter-wave band by directing the beam toward the thoracic region [23], thereby achieving minimally invasive electromagnetic energy delivery [24] and enhanced clutter suppression in the near field. Additional applications include near-field millimeter-wave communications [25], near-field synthetic aperture radar (SAR) illumination [26], wireless power transfer (WPT) [27,28,29], and high-power microwave (HPM) systems for counter-UAV operations [30], where controlled near-field energy delivery over finite target regions is required. Since these applications typically do not require real-time optimization, the phase distributions can be precomputed offline for each operational scenario.

Future research will focus on extending the proposed framework to fully two-dimensional experimental validation, conformal and volumetric array geometries, non-planar target surfaces, adaptive beam synthesis for dynamically changing scenarios, and computationally efficient optimization algorithms for large-scale near-field beam synthesis, including multilevel methods for accelerating Fresnel-region computations [31].

## Figures and Tables

**Table 1:** Focusing phase values from the simulation.

Denotation	Phase Value_1 (deg)	Value_1 (deg)	Phase Value_2 (deg)	Value_2 (deg)
Element 1	348.75	112.5	185.625	73.125
Element 2	286.875	50.625	151.875	39.375
Element 3	270	33.75	123.75	11.25
Element 4	236.25	0	118.125	5.625
Element 5	247.5	11.25	112.5	0
Element 6	258.75	22.5	129.375	16.875
Element 7	292.5	56.25	151.875	39.375
Element 8	343.125	106.875	185.625	73.125

**Table 2:** Design requirements.

Denotation	Symbol	Value
Operating frequencies	fc	2.3–2.5 GHz
Dielectric substrate	eps_r	FR4–4.3
Thickness	t	0.035 mm
Height of the dielectric substrate	h_sub	1 mm
Return loss	S11	<−10 dB
Impedance of the antenna	dipole	73 Ohms
Beamwidth	BW	60 deg

**Table 3:** Focusing phase values of set 1 ($x_{f}=1\;m$).

Denotation	Required Phase (deg)	Measured Phase (deg)	Electric Length (mm)	Relative Phase (deg)	Error Phase (deg)
Element 1	112.5	152.9	51.0	110.9	−1.6
Element 2	50.625	95.2	40.0	53.2	2.6
Element 3	33.75	75.9	35.0	33.9	0.1
Element 4	0	42	29.0	0	0
Element 5	11.25	54.4	31.5	12.4	1.1
Element 6	22.5	69.9	34.0	24.9	2.4
Element 7	56.25	97.2	40.5	55.2	−1.1
Element 8	106.875	147.8	50.1	105.8	−1.1

**Table 4:** Focusing phase values of set 2 ($x_{f}=1.5\;m$).

Denotation	Required Phase (deg)	Measured Phase (deg)	Electric Length (mm)	Relative Phase (deg)	Error Phase (deg)
Element 1	73.1	118.5	43.5	71.2	−1.9
Element 2	39.4	87	37.0	39.7	0.3
Element 3	11.3	58	32.0	10.7	−0.6
Element 4	5.6	53.2	30.5	5.9	0.3
Element 5	0	47.3	29.5	0	0
Element 6	16.9	64.2	33.0	16.9	0
Element 7	39.4	84.1	37.0	36.8	−2.6
Element 8	73.1	117.9	43.5	70.6	−2.5

**Table 5:** Focusing phase values of sets within the feeding network.

Denotation	Required Phase (deg)	Measured Phase (deg)	Relative Phase (deg)	Error Phase (deg)
Element 1	−73.1	−27.6	−65.4	7.7
Element 2	−39.4	1.6	−36.2	3.2
Element 3	−11.3	28.7	−9.1	2.2
Element 4	5.6	38.5	0.7	4.9
Element 5	0	37.8	0	0
Element 6	−16.9	22.7	−15.1	1.8
Element 7	−39.4	2.1	−35.7	3.7
Element 8	−73.1	−30.9	−68.7	4.4

## Data Availability

All data included in this study are available upon request by contacting the corresponding author.

## Appendix A

**Table A1:** Dipole results.

Denotation	Symbol	Value
50 ohms transition Line length	L_50_ohms	5 mm
50 ohms transition Line width	W_50_ohms	1.9 mm
Rectangular ground width	W_ground	35 mm
Quarter wave transformer length	L_quarter	24.25 mm
Quarter wave transformer width	W_quarter	0.6 mm
Dipole width	W_driver	0.6 mm
Half Dipole length	L_driver	25.77 mm

This antenna’s element was designed with balun feeding [32], simulated, and tested, resulting in the performance shown in Figure A1:
